# The Tribaloy T-800 Coatings Deposited by Laser Engineered Net Shaping (LENS^TM^)

**DOI:** 10.3390/ma12091366

**Published:** 2019-04-26

**Authors:** Tomasz Durejko, Magdalena Łazińska, Julita Dworecka-Wójcik, Stanisław Lipiński, Robert A. Varin, Tomasz Czujko

**Affiliations:** 1 Department of Advanced Materials and Technologies, Military University of Technology, 2 Gen. Sylwestra Kaliskiego Street, 00-908 Warsaw, Poland; tomasz.durejko@wat.edu.pl (T.D.); magdalena.lazinska@wat.edu.pl (M.Ł.); stanislaw.lipinski@wat.edu.pl (S.L.); 2 Department of Mechanical and Mechatronics Engineering, University of Waterloo, Waterloo, ON N2L 3G1, Canada; robert.varin@uwaterloo.ca

**Keywords:** tribaloy-type alloy, CoCrMoSi alloy coatings, T-800 alloy, Laves phase, Laser Engineered Net Shaping (LENS^TM^)

## Abstract

A Tribaloy family of alloys (CoMoCrSi) are characterized by a substantial resistance to wear and corrosion within a wide range of temperatures. These properties are a direct result of their microstructure including the presence of Laves phase in varying proportions. Tribaloy T-800 exhibits the highest content of Laves phase of all other commercial Tribaloy alloys, which provides high hardness and wear resistance. On the other hand, a large content of the Laves phase brings about a high sensitivity to brittle fracture of this alloy. The main objective of this work was a development of the Tribaloy T-800 coatings on the Ni-based superalloy substrate (RENE 77), which employs a Laser Engineered Net Shaping (LENS^TM^) technique. Technological limitations in this process are susceptibility of T-800 to brittle fracture as well as significant thermal stresses due to rapid cooling, which is an inherent attribute of laser techniques. Therefore, in this work, a number of steps that optimized the LENS^TM^ process and improved the metallurgical soundness of coatings are presented. Employing volume and local substrate pre-heating resulted in the formation of high quality coatings devoid of cracks and flaws.

## 1. Introduction

Nickel-based (Ni-based) superalloys exhibit a combination of high strength and corrosion/oxidation resistance at elevated temperatures. 

These virtues make the Ni-based superalloys widely used for producing components of various systems working at extreme service conditions such as gas turbines in jet engines, pumps, and pipes for chemical and petrochemical industries and rocket engines. A long service at extreme service conditions eventually leads to severe wear of Ni-based superalloy parts, particularly at the surface layer, which limits their useful service life [1]. Therefore, new materials and technologies are being sought, which could improve efficiency and service life of highly stressed components working at high temperatures. In order to regenerate those components and bring them to the original condition, at least partially, protective coatings are applied to extend their useful service life [2,3]. The regenerative coatings also allow us to control certain properties like the increase of corrosion/oxidation and wear resistance. These coatings also prevent the effects of rapid temperature fluctuations. For the components made of Ni-based superalloys that work under severe wear conditions, the improvement of their wear resistance is of paramount importance. A good solution in this case is applying a coating that would be more wear resistance than the substrate being simultaneously highly corrosion/oxidation resistant. Suitable materials are quite commonly used such as cobalt (Co)-based alloys that have trademark names Stellite and Tribaloy (trademarks of the Deloro Stellite Company, Inc., Koblenz, Germany). These alloys are characterized by high strength, hardness, corrosion/oxidation, and wear resistance at a wide range of temperatures [4]. These properties are due to a unique microstructure, which consists of hard particles (either carbides or intermetallic phases) embedded in the Co solid solution matrix. According to the producer specifications, the Stellite alloys are recommended for components requiring corrosion/oxidation, erosion, and wear resistance at temperatures up to 800 °C. In turn, the Tribaloy alloys are recommended for the components requiring wear and corrosion/oxidation resistance at high temperatures, particularly, for applications where a direct lubrication is impossible [5]. 

Since Ni-based alloy components are frequently in service at temperatures exceeding 800 °C, it is justified to coat them with Tribaloy coatings since it was already implemented by Rolls Royce for turbine blades [6].

The principal alloying element of the Tribaloy alloys is Co in addition to molybdenum (Mo), chromium (Cr), and silicon (Si). Their properties depend on the content of each alloying element.

The most common commercial Co-based Tribaloy alloys are T-400, T-800, and T-900 [7]. Other alloys are T-400C and T-401, whose compositions are modifications of a base alloy T-400 [8,9,10]. The Tribaloys are usually hypereutectic (except T-401) and have a cast microstructure, which consists of hard, primary intermetallic Laves phases (about 40–60 vol.%) and are uniformly distributed in the eutectic matrix (fine intermetallic dispersoids within the Co solid solution) [11,12]. In Tribaloys, the Laves phases are intermetallic phases of the C-14-type (MgZn_2_) like Co_3_Mo_2_Si and/or CoMoSi. They are characterized by high hardness approaching 1000-1200 HV [8] and high melting point of about 1560 °C [10,13]. The fraction of these phases in Tribaloy alloys is determined by a large content of Mo and Si as compared to other Co-based alloys like Stellite alloys. A minimal amount of carbon in Tribaloy alloys prevents formation of the M_7_C_3_-type precipitates that are found in Stellite alloys. 

Tribaloy T-800, which contains the largest content of Laves phase at about 60 vol.% in its microstructure, exhibits the highest hardness while a T-400 alloy contains only about 40 vol.% of the Laves phase [12]. In addition, a large content of Laves phase in T-800 provides high adhesive and abrasive wear resistance while it, simultaneously, reduces plasticity and impacts toughness at an ambient temperature. Furthermore, T-800 is also characterized by increased oxidation resistance at a wide range of temperatures attributable to its high chromium content (nearly twice as much as that in T-400).

Initially, the Tribaloy coatings were deposited by thermal processing such as thermal spraying [14,15]. Due to a narrow heating zone, those methods did not provide a good adherence of the deposited layer via metallurgical bonding with the substrate [16]. Therefore, more recently, reports in the literature state the laser techniques employed for the Tribaloy coatings [17]. It is expected that these processes would result in coatings with good metallurgical properties, fine-grained with only minor structural modifications within the deposit-substrate volume, and precisely mirroring the desired shape.

For T-800, the presence of Laves phases in a large quantity guarantees high hardness and wear resistance, although a high content of this brittle phase is simultaneously a drawback, since it favors the brittle crack formation and propagation. That inherent propensity to brittle behavior makes the coating processes more difficult since cracking must be avoided especially in small components [18,19]. The propensity of brittle fracture of Tribaloy T-800 is a problem for laser techniques for which substantial thermal stresses arise during rapid cooling, which exacerbate the propensity for brittle fracture. New Tribaloy alloys have been implemented with the objective of ameliorating this problem. A modification of the base composition results in an improvement of plasticity, resistance to brittle fracture, and, simultaneously, lowers hardness. For example, T-900 having an increased Ni content (a lower content of Co and Mo) has better plasticity and fracture resistance than T-800. Alternatively, alloying of T-800 with iron (Fe) can also improve some properties. If the Fe content exceeds 10 wt.%, the microstructure undergoes a substantial alteration such that dendrites are refined, Laves phases disappear, and the alloy becomes more fracture-resistant while its hardness and wear resistance are lowered [17]. 

Navas et al. [18] reported that an intelligent choice of processing parameters can also alleviate the problems described above. The technology of Laser Cladding permitted us to obtain good quality T-800 coatings on an AISI 304 substrate. The obtained coating exhibited a high hardness about 850 HV0.3 and measurably improved wear resistance of the substrate [18]. In addition, in order to reduce thermal stresses in the coating, which can be a source of brittle cracking, it could be beneficial to pre-heat the substrate just before depositing the coating [19,20]. 

Due to its characteristics, T-800 seems to be a very attractive alloy for the components exposed to high service temperatures and wear conditions, like turbine blades. In these applications, besides wear resistance, a substantial oxidation resistance is also required. Yuduo et al. [21] reported that T-800 has high oxidation resistance at elevated temperatures, which can be additionally enhanced by alloying with 4 wt.% rhenium (Re). 

In this work, an attempt is made to investigate varying processing parameters for producing coatings of Tribaloy T-800 on the Ni-based superalloy, RENE 77, substrate. In order to obtain metallurgically sound deposits, devoid of microcracks and flaws, exhibiting a gradual distribution in the chemical composition and microhardness at the interface deposit-substrate, the coatings were produced by the LENS^TM^ (Laser Engineered Net Shaping) technique. Due to local substrate heating, this technique minimizes the heat affected zone. This, in turn, reduces “thermal distortions” and the amount of substrate material in the deposit. In addition, LENS^TM^ allows depositing coatings with controlled thickness on any randomly selected area of the substrate.

## 2. Materials and Methods

### 2.1. Characterization of the Initial Powder

**Figure 1:**
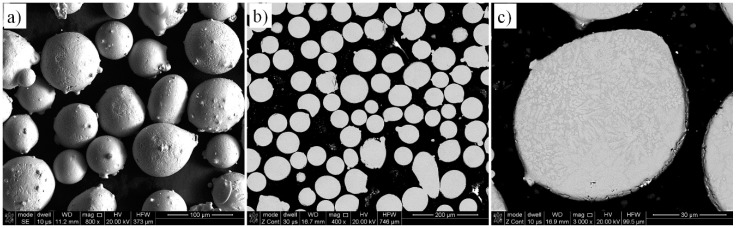
Initial T-800 powder: (**a**) 3D view, (**b**) metallographic cross-section, and (**c**) microstructure of the particle.

The initial powder was Tribaloy T-800 provided by LPW Technology Ltd. (Widnes, UK). The powder particles analyzed with an electron microscope Quanta 3G FEM Dual Beam (FEI, Hillsboro, OR, USA) shows a spherical morphology (Figure 1a). A powder cross section reveals a miniscule porosity (Figure 1b) and a dendritic microstructure within the particles (Figure 1c). 

**Figure 2:**
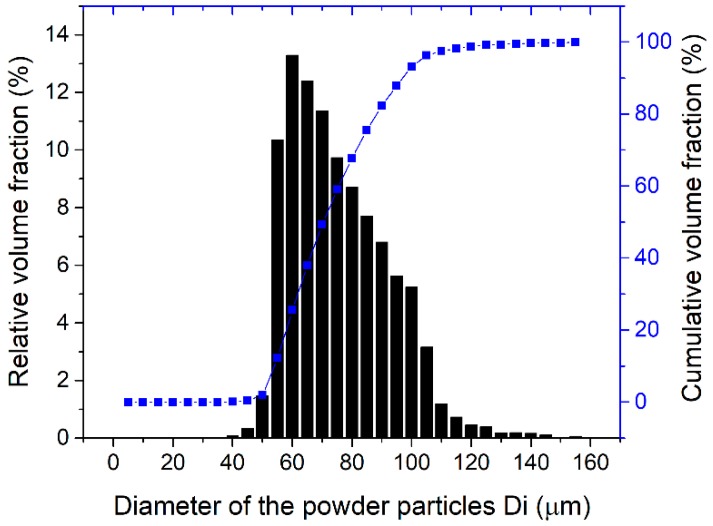
Particle size distribution of the T-800 powder employed in this work.

Chemical composition measurements by energy dispersive X-ray spectroscopy (EDAX, Mahwah, NJ, USA) confirmed the nominal chemical composition from the producer (Table 1). The particle size distribution analysis showed that about 99% particle sizes was within the range of 44 to 150 μm (Cumulative Volume Fraction in Figure 2). The T-800 powder has high microhardness of 1180 HV0.1.

### 2.2. Experimental Procedures

Deposits of the T-800 alloy were produced using a Laser Engineered Net Shaping (LENS^TM^) technique. This is one of the large family 3D printing techniques, whose great advantage is a production of components with pre-determined geometries and nearly any shape [22,23,24].

Currently, LENS is used for manufacturing components made from a wide range of materials, like stainless steels, titanium, nickel, or cobalt-based alloys [25,26]. A control of process parameters such as the powder flow rate, the laser power, and the travel of the working table allows us to obtain a desired microstructure of the components and modification of their surfaces, which increases wear and corrosion resistance [27]. It can also be employed in the regeneration of worn out components [28].

Fabrication of the T-800 coatings was carried out using the LENS 850-R system (Optomec, Albuquerque, NM, USA). It contains a movable table (moving along the X and Y axes), on which a substrate is placed. Inside the work chamber, there is a laser and nozzles supplying the powder under an argon protective atmosphere. Powder is directed into the laser beam focus zone where it is melted and deposited on the substrate. The entire process is computer controlled and results in a controlled thickness of a deposited coating. 

The coatings were deposited on the polished and sand blasted RENE 77 alloy substrate with the composition in Table 2. The dimensions of the substrate plates were as follows: 35 mm × 15 mm × 13 mm (length × width × height).

The dimensions of the deposit made by the controlled geometry LENS technique are as follows: width 10 mm, length 10 mm, and height 2.5 mm.

The metallurgical soundness of each deposit was investigated by an optical (Nikon Eclipse MA2000, Amsterdam, The Netherlands) and stero (Nikon SMZ1500, Amsterdam, The Netherlands) microscopes. Both surface and cross-sectional metallographic samples were investigated. Porosity of deposits was assessed by stereological image analysis and expressed as a ratio of the total area of pores to the total area of selected surface, according to the following formula:

(1) $$P=\frac{S_{p}}{S_{cz}}\times 100\%$$

where: P—porosity (%); Sp—the total pore area (μm^2^); Scz—the total area of selected surface (μm^2^).

X-ray computed microtomography (μCT) (Nikon/METRIS XT H 225 ST, Leuven, Belgium) was employed on selected deposits to assess the presence of flaws and cracks in the material. 

Microstructural observations and chemical analysis (point, linear, and element mapping) were carried out with a scanning electron microsope FEI Quanta 3G FEM Dual Beam (Hillsboro, OR, USA) equipped with an EDS attachment. Phase analysis was carried out in a Rigaku ULTIMA IV diffractometer (Neu-Isenburg, Germany) utilizing CoK_α_ radiation ranging from 20 to 140° in 2θ with a step size of 0.02° and exposure time of about 3s. An acceleration voltage of 40 kV and 20 mA current were applied.

The Vickers microhardness profile in the substrate-coating was investigated with a microhardness tester Shimadzu HMV (Duisburg, Germany) under a load of 100 G and 10-s dwell time. The indents were made every 100 µm starting from the substrate side.

## 3. Results and Discussion

### 3.1. Selection of LENS Parameters

For coating fabrication by the LENS technique, the following process parameters were always fixed: carrier gas flow in a powder supply unit and in a central nozzle was 4 and 25 l/min, respectively. The content of oxygen and water vapor in the working chamber was controlled during the process at the level of 22.7 and 8 ppm, respectively. The thickness of each deposited layer was 0.5 mm. In order to find out the optimal LENS process parameters, a number of trials was carried out where the laser power, powder feeding rate, laser head travel rate, and substrate temperature were varied accordingly. Table 3 shows the working parameters of fabricated coatings. The powder feeding rate was 7.6 g/min for sample 1, 5.2 g/min for sample 6, and 4.6 g/min for other samples. The laser head travel rate ranged from 4 to 16 mm/s. Laser power was within a range of 300 to 500 W even though, for the first two layers in samples 15–18, the laser power was 300 W and the remaining layers were fabricated with the power of 500 W. In addition, the substrate temperature was also regulated. Some coatings were deposited at ambient temperature (samples 1–3) and others after pre-heating the working table to 300 °C. For selected coatings besides pre-heating of the working table, the surface was heated by a laser with a power within a range of 150 to 300 W. 

**Figure 3:**
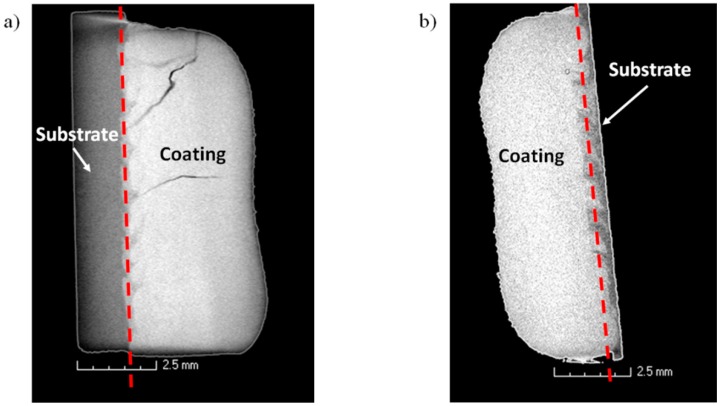
Microfocus X-ray computer tomography image of the LENS-fabricated samples: (**a**) nr 2 and (**b**) nr 18.

Microscopic observations (not shown) revealed that the coatings fabricated without surface and/or volume substrate pre-heating contained numerous surface and volume cracks and flaws that, most likely, were generated by thermal stresses due to a rapid cooling rate. Those coatings that were fabricated with the substrate volume pre-heating up to 300 °C showed a greatly reduced number of cracks and, in the case of additional point laser heating, the cracks disappeared. Figure 3a,b shows an X-ray computer tomography image for a volume cracked sample and the one which is metallurgically sound, respectively.

**Figure 4:**
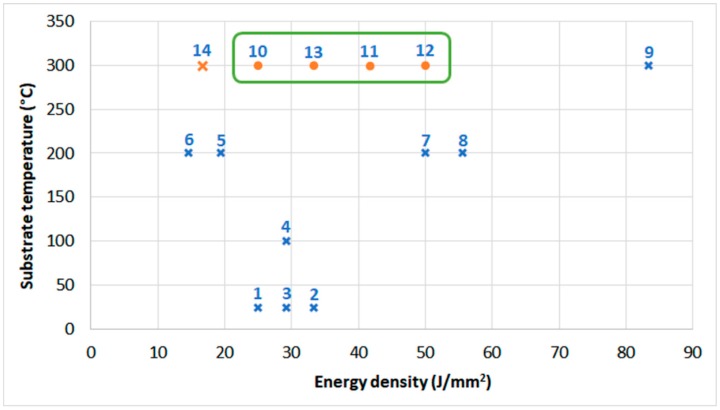
Plot of dependence between substrate temperature and energy density (for samples 1–9, the energy density was calculated using the laser power employed in the depositing process—marked in blue. For samples 10–14, the energy density was calculated using the laser power employed for the substrate heating—marked in orange). The X marks indicate samples with cracks.

Figure 4 shows dependence between substrate temperature and energy density. For samples 1–9, the energy density was calculated using the laser power employed in the depositing process. For samples 10–14, the energy density was calculated using the laser power employed for the substrate heating since it was different than the power for the depositing process. The X marks samples with cracks. 

Figure 4 shows that it is feasible to obtain metallurgically sound microstructure of Tribaloy T-800 coatings (no cracks) when the substrate is furnace pre-heated to 300 °C with the additional substrate point laser heating if the energy density is within the range of 25 to 50 J/mm^2^. Lower energy density results in coatings’ cracking. However, since the geometrical shape of fabricated coatings diverged from the required geometrical model, the coating process was further modified. 

The pre-determined geometrical coating dimensions were 10 mm × 10 mm × 2.5 mm with the narrowest dilution zone possible in order to avoid any chemical content changes of the deposit and its properties. Coatings 1–14 diverged greatly from the desired dimensions, which prompted us to change the parameters of fabrication (samples 15–18). The new processes was based on the LENS parameters for sample 13. The laser power was modified such that, when depositing of the first two layers, the power was 300 or 400 W with a further increase to 500 W for the next three to five layers.

Microscopic observations of the cross-sections of samples 13–18 were used to find out the characteristic dimensions of the coatings such as the height, width, dilution zone, and porosity, which are shown in Table 4.

The measurements indicate that the characteristic dimensions of Tribaloy T-800 coatings strongly depend on the processing parameters. The coating height nearest the required one (2.5 mm) is exhibited by sample 17, while sample 14 shows depth near the required one (10 mm).

Sample 13 exhibits the largest dilution zone approaching nearly 0.9 mm, while sample 15 has the smallest dilution zone of 0.07 mm, which was fabricated by varying laser power of 300 W for the first two layers and 500 W for the remaining layers. Apparently, using a lower laser power for depositing the first few layers, is beneficial for getting a small dilution zone. Table 4 also includes the results of porosity measurements, which show that samples 15 and 16 has high porosity of 2.6% and 5.3%, respectibvely. The remaining samples have porosity values less than 1%.

Taking into account the results in Table 3 and Table 4, the optimal combination of processing parameters, which result in dimensions close to the required ones is met for sample 18.

### 3.2. Characterization of Obtained Coatings

#### 3.2.1. Microstructure

**Figure 5:**
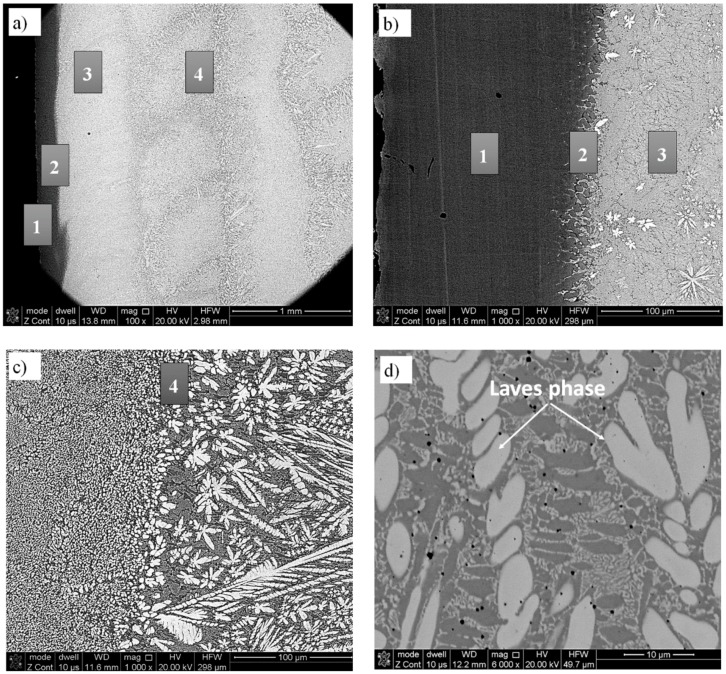
The SEM images of the microstructure of T-800 coating—specimen number 18. Coating/substrate interface (**a**,**b**) and coatings (**c**,**d**).

The microstructure of metallurgically sound sample 18 without cracks and with the lowest porosity (Table 3 and Table 4) was investigated by scanning electron microscopy. The SEM images of the areas on the substrate-deposit interface are shown in Figure 5.

Figure 5a,b show the microstructure of the interface between the deposit and substrate while Figure 5c,d show the microstructure of the deposit bulk. 

Figure 5a shows a few areas numbered 1–4. Area #1 is a substrate, area #2 is a deposit melted into the substrate, area #3 is the first deposit layer, and area #4 is the deposit. Higher magnifications of these areas are shown in Figure 5b,c. In addition, in Figure 5c, the microstructure at the cross section of two layers that were deposited transverse to one another, as indicated by the orientation of the bright phase, is observed. Figure 5c,d clearly show a dendritic microstructure of the deposit. Some characteristic phases are observed in Figure 5d at higher magnification.

**Figure 6:**
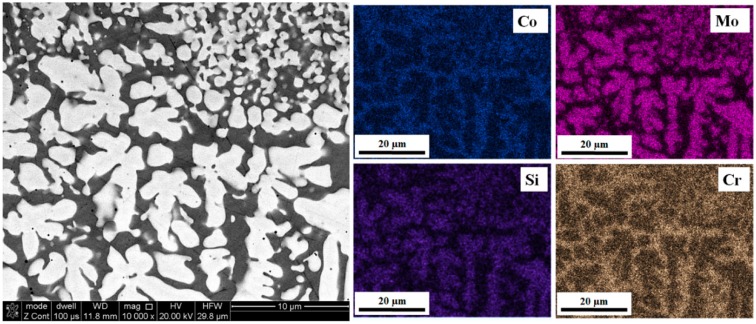
Elemental distribution maps for the T-800 coatings.

Elemental distribution maps in Figure 6 show the presence of two phases. Light dendrites are rich in Mo and silicon Si while the interdendritic spaces contain mainly Co and Cr.

**Figure 7:**
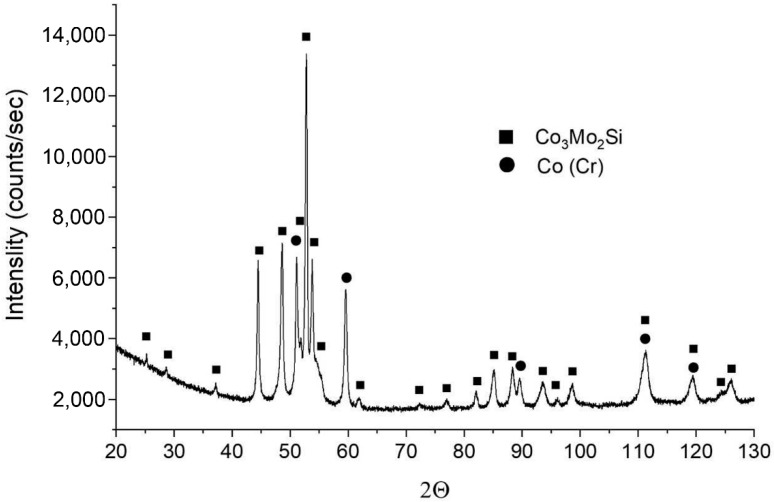
XRD patterns of the T-800 coatings obtained using the LENS technique.

An example of the X-ray diffraction pattern in Figure 7 confirms the presence of two phases: Co_3_Mo_2_Si (#01-082-6068 in DHN PDF 4 database) and a Co-based one (#01-071-4238 in DHN PDF 4 database).

**Figure 8:**
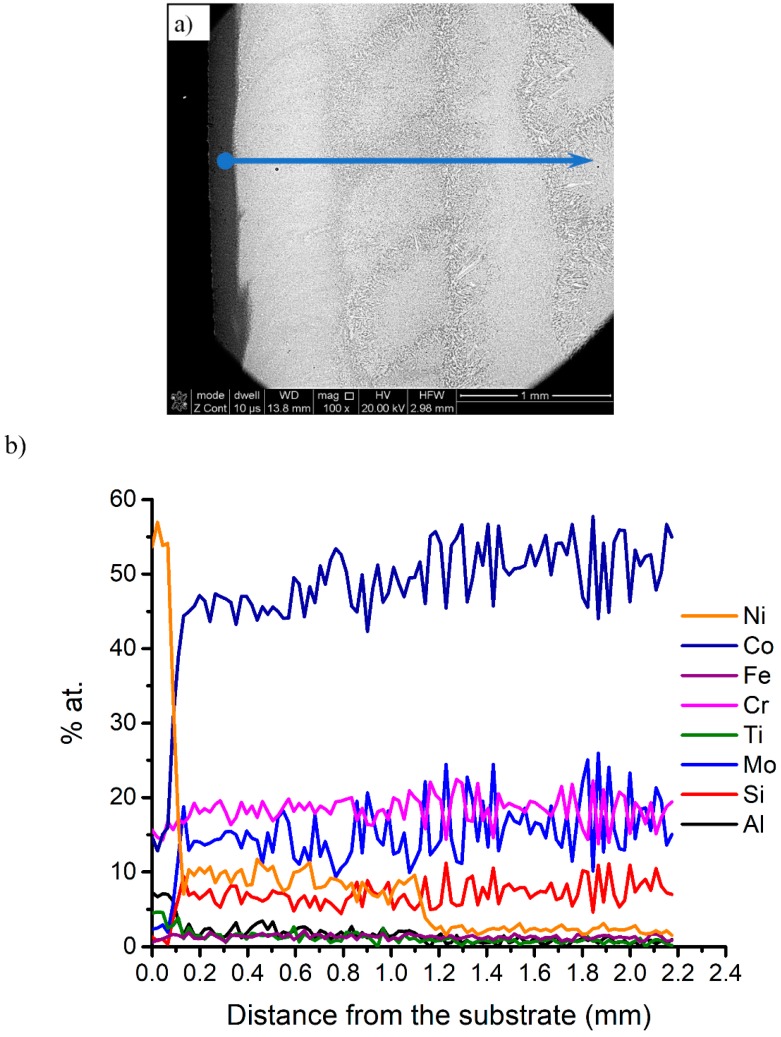
The results of the EDS linear chemical composition analysis of the T-800 coatings specimen 18: (**a**) a macrograph of the deposit with the arrow indicating the direction of linear EDS analysis. (**b**) The chemical content of the elements as a function of the distance from the substrate.

In order to find out a chemical composition profile at the substrate-deposit interface, a linear EDS analysis was carried out. Ideally, the interface should exhibit a continuous compositional change. The EDS analysis started from the substrate toward the deposit (Figure 8a) and the results in Figure 8b indicate a rapid change in the content of Ni, Co, Mo, and Cr. As expected, with increasing distance from the substrate, the content of Ti decreases and traces of Si start appearing. The Ni content initially rapidly drops at the substrate-deposit interface then levels up and, at the distance of 1 mm from the interface, decreases to nearly zero. Within a 1-mm distance from the interface, the dominant elements are Co, Mo, Cr, i, and Si, which correspond well to the original composition of the initial powder.

#### 3.2.2. Microhardness

**Figure 9:**
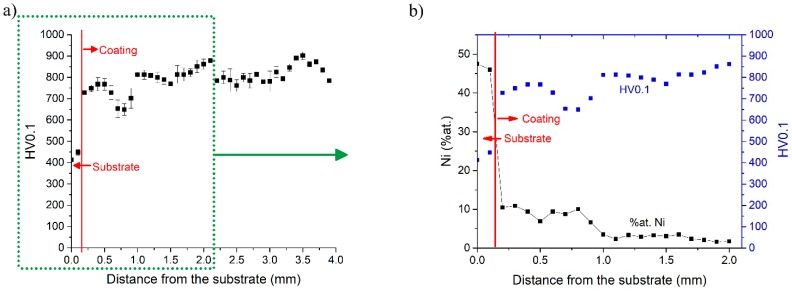
(**a**) Microhardness distribution of the T-800 coatings deposited on RENE 77 using the LENS process. (**b**) Microhardness distribution and Ni content in sample 18.

As shown in Figure 9a, the microhardness values show a drop (Figure 9a) at a distance of about 750 µm from the substrate, which, as shown in Figure 9b, is due to a local increase in the Ni content at the same distance. Figure 9b also shows that, at a distance of about 1 mm from the substrate, the Ni content drops to 1–2 at.% while microhardness increases to the 800–900 HV0.1 range and then stabilizes approximately at the same level.

The average microhardness of the RENE 77 substrate is about 450 HV0.1 while the microhardness of the deposited Tribaloy T-800 coating is nearly two-fold higher. However, the microhardness of the Tribaloy T-800 coating is nearly 300 HV0.1 lower than that of the initial Tribaloy T-800 powder (1180 HV0.1). The difference in hardness between the T-800 powder and the coating made of this powder is due to the cooling rate used during the manufacturing process. The cooling rate during powder atomization is much higher than the cooling rate used in the LENS process (for example: cooling rate of argon-gas atomized nickel—10^5^–10^6^ K/s, LENS process—10^3^ K/s).

### 3.3. Discussion

The present work clearly shows that laser techniques and, in particular, the LENS technique, are capable of fabricating the Tribaloy T-800 coatings on the RENE 77 substrate. Testing of various fabrication process parameters has clearly shown that, in order to obtain a metallurgically sound coating, without cracking, a substrate temperature must be judiciously adjusted. In those variants of the process without substarte pre-heating, various surface and volume cracks and flaws were always formed in the deposits, as reported by Diaz [19]. Furthermore, Przybyłowicz i Kusinski [29] recommended heating a Ni-based superalloy substrate (IN718) to 500 °C in order to eliminate cracking in the Tribaloy T-400 coating. In the present work for the T-800—RENE 77 materials, a sole substrate volume pre-heating was not completely effective since cracks still appeared in the sample. A better solution was a combination of substrate volume pre-heating with a point laser heating having energy density within a range of 25 to 50 J/mm^2^. Therefore, by heating the substrate to a temperature of about 550 °C (by heating the working table and laser preheating) and by changing energy density during the fabrication of the coating, we achieve a coating with a good geometrical quality, which exhibits a minimal dilution zone with the substrate and is devoid of cracks. 

In this work, we eliminated cracks by pre-heating the substrate. Other solutions are known in the literature to help avoid cracks, such as the ones using intermediate layers [30].

A great advantage of the LENS technique is the possibility of depositing coatings with pre-determined dimensions. Fabrication processes have shown that, through an adjustment of the process parameters, it is feasible to fabricate a metallurgically sound coating, without porosity/cracking and with good adhesion to the substrate with a pre-determined geometry.

A Tribaloy T-800 coating fabricated in the present work is characterized metallurgically by the microstructure typical for the commercial Tribaloy alloys, in which two phases are present. The first phase is an intermetallic Laves phase, which contains predominantly Co, Mo i Si (Co_3_Mo_2_Si), embedded in the Co solid solution matrix. Navas et al. also reported a similar microstructure [18].

The results of the measurements of linear chemical composition and microhardness changes in the deposited sample show a fluctuation of elemental distribution of alloying elements, in particular Ni, from the substrate to the deposit. The effect of the Ni distribution content on microhardness is still observed up to about 1 mm distance from the substrate. 

The Tribaloy T-800 coatings on the RENE 77 substrate increase microhardness of the working surface by about 400 HV with respect to the microhardness of the RENE 77 substrate. 

## 4. Conclusions

The research carried out in this work shows that it is feasible to fabricate the metallurgically sound Tribaloy T-800 coatings, devoid of cracks and flaws, on the RENE 77 substrate using the LENS technique. It has been found that the technological coating process parameters greatly influence the metallurgical soundness of coatings. A principal process parameter influencing quality is the substrate temperature. Depositing the Tribaloy T-800 coatings using the LENS technique should be carried out on the pre-heated surface, which greatly reduces thermal stresses and prevents cracking and flaw formation within the cross section of the deposit. Microscopic observations confirm a formation of the dual phase microstructure, which is typical for the commercial Tribaloy alloys. These alloys consist of Laves phase dendrites embedded in the Co solid solution matrix. Linear chemical analysis combined with microhardness measurements indicate chemical composition fluctuations, predominantly Ni, at the interface substrate-coating. Microhardness increases nearly two-fold toward the deposit.

## Figures and Tables

**Table 1:** Chemical composition of T-800 powder.

T-800 Powder						
Element Wt.%	Co	Mo	Cr	Si	Fe	Ni
**Spherical particle**	Bal.	27	16.9	3.0	0.5	0.8
**Nominal (manufacturer)**	Bal.	27–30	16.5–18.5	3–3.8	Fe + Ni max 3	

**Table 2:** Chemical composition of RENE 77 alloy.

RENE 77 Alloy						
Element	Ni	Cr	Ti	Mo	Al	Co
**wt.%**	bal.	15.2	3.3	3.8	3.7	16.4

**Table 3:** The set of the LENS process parameters used during deposition of T-800 coatings.

Sample Number	Energy Density * (J/mm^2^)	Powder Feeding Rate (g/min)	Substrate Temperature	Remarks
**1**	25	7.6	Ambient temperature	Numerous surface and volume cracks
**2**	33.3	4.6		
**3**	29.2	4.6		
**4**	29.2	4.6	Pre-heating working table to 100 °C	
**5**	19.4	4.6	Pre-heating working table to 200 °C	
**6**	14.6	5.2	Pre-heating working table to 200 °C	
**7**	50.0	4.6	Pre-heating working table to 200 °C	
**8**	55.6	4.6	Pre-heating working table to 200 °C	
**9**	83.3	4.6	Pre-heating working table to 300 °C	
**10**	83.3	4.6	Pre-heating working table to 300 °C with a laser of 150 W (energy density: 25 J/mm^2^)	No surface cracks. Coating dimensions diverge from the assumed geometrical model.
**11**	83.3	4.6	Pre-heating working table to 300 °C with a laser of 250 W (energy density: 41.7 J/mm^2^)	
**12**	83.3	4.6	Pre-heating working table to 300 °C with a laser of 300 W (energy density: 50 J/mm^2^)	
**13**	83.3	4.6	Pre-heating working table to 300 °C with a laser of 200 W (energy density: 33.3 J/mm^2^)	No cracks
**14**	41.7	4.6	Pre-heating working table to 300 °C with a laser of 200 W (energy density: 16.7 J/mm^2^)	Volume cracks
**15**	Two layers 50, the others 83.3	4.6	Pre-heating working table to 300 °C with a laser of 200 W (final temperature—around 550 °C)	Volume cracks and porosity
16 **	Two layers 50, the others 83.3	4.6		No cracks, porosity
**17**	Two layers 50, the others 83.3	4.6		Volume cracks
18 **	Two layers 66.7, the others 83.3	4.6		No cracks

* Energy delivery per unit area of material: E = P/(2r_b_V_beam_), where P—laser power (W), r_b_—beam radius (mm), and V_beam_—scan speed (mm/s). Applied beam diameter = 1.5 mm. ** The samples were made with the Hatch shrink = 0.3 mm.

**Table 4:** Characteristic dimensions and porosity of T-800 coatings deposited with different LENS parameters.

Coatings T-800				
Sample Number	Height (mm)	Width (mm)	Dilution Zone (mm)	Porosity (%)
**13**	3.4	11.6	0.88	0.68
**14**	3.9	**10.0**	0.23	0.85
**15**	3.8	10.7	**0.07**	2.60
**16**	3.1	10.9	0.12	5.30
**17**	**2.8**	11.4	0.25	0.67
**18**	3.2	10.8	0.10	0.54
